# Embryonal Rhabdomyosarcoma: The Diagnostic Dilemma of a Rare Laryngeal Malignancy

**DOI:** 10.7759/cureus.26959

**Published:** 2022-07-18

**Authors:** Farahin Rosdi, Najihah Hanim Asmi, How Kit Thong, Primuharsa Putra Sabir Husin Athar, Aifaa Abdul Manan

**Affiliations:** 1 Otolaryngology - Head and Neck Surgery, Hospital Shah Alam, Shah Alam, MYS; 2 Otolaryngology - Head and Neck Surgery, Hospital Sultan Haji Ahmad Shah, Temerloh, MYS; 3 Otolaryngology - Head and Neck Surgery, Hospital Sultan Ismail, Johor Bahru, MYS; 4 Otolaryngology - Head and Neck Surgery, Kumpulan Perubatan Johor (KPJ) Healthcare University College, Nilai, MYS; 5 Otolaryngology - Head and Neck Surgery, Kumpulan Perubatan Johor (KPJ) Seremban Specialist Hospital and KPJ Healthcare University College, Seremban, MYS

**Keywords:** pediatrics, pediatric laryngology, autoimmune disease, laryngeal carcinoma, laryngeal embryonal rhabdomyosarcoma

## Abstract

The larynx and the remaining components of the upper aerodigestive tract collectively play an important role in undertaking respiration, phonation, and deglutition. Therefore, a variety of pathologies can present with similar symptoms. Systemic diseases, such as rheumatoid arthritis and relapsing polychondritis, may also manifest with laryngeal symptoms and findings, whereas rare pathologies may present with vague presentations. Such scenarios may be attributed to the consequent medical confusion and dilemma in reaching an accurate diagnosis. In this case report, an 11-year-old male presented with airway compromise symptoms, which were later identified and diagnosed as laryngeal embryonal rhabdomyosarcoma.

## Introduction

Rhabdomyosarcoma is a malignant mesenchymal neoplasm and the most commonly observed soft tissue sarcoma in the children and adolescent age groups. The tumor often resembles the muscle cells found in 7- to 10-day-old embryos. The four principal histological variants of rhabdomyosarcoma consist of embryonal, alveolar, pleomorphic, and botryoid, whereby, in general, the embryonal and pleomorphic subtypes occur in the younger and older population, respectively [1,2]. Their clinical presentation may be distinguished from other laryngeal malignancies, in which some may present with subtle clinical signs. Similar instances are also seen in autoimmune diseases, which may underline indistinguishable presentation but can be diagnostically supported and proven using certain benchmark antibodies, such as antinuclear antibodies (ANA).

## Case presentation

We describe a case of an 11-year-old male with underlying allergic rhinitis, who initially presented with an acute episode of cough and shortness of breath. During the first presentation, he was treated as a case of acute exacerbation of asthma and admitted to the intensive unit care (ICU) for noninvasive ventilation support. However, no intubation was needed during that episode. Upon his discharge, he continued to complain of coughing at home but did not have any nocturnal or exercise- or cold-induced acute exacerbation of asthma. About two months later, he developed another episode of shortness of breath, but there was no history of fever, sore throat, or other symptoms of inflammation. During the assessment, he was noted to be in respiratory distress with severe tachypnea. The attending physician faced severe difficulty during intubation. Subsequent video laryngoscopy revealed severe upper airway obstruction at the subglottic region, which led to the diagnosis of severe subglottic stenosis.

**Figure 1:**
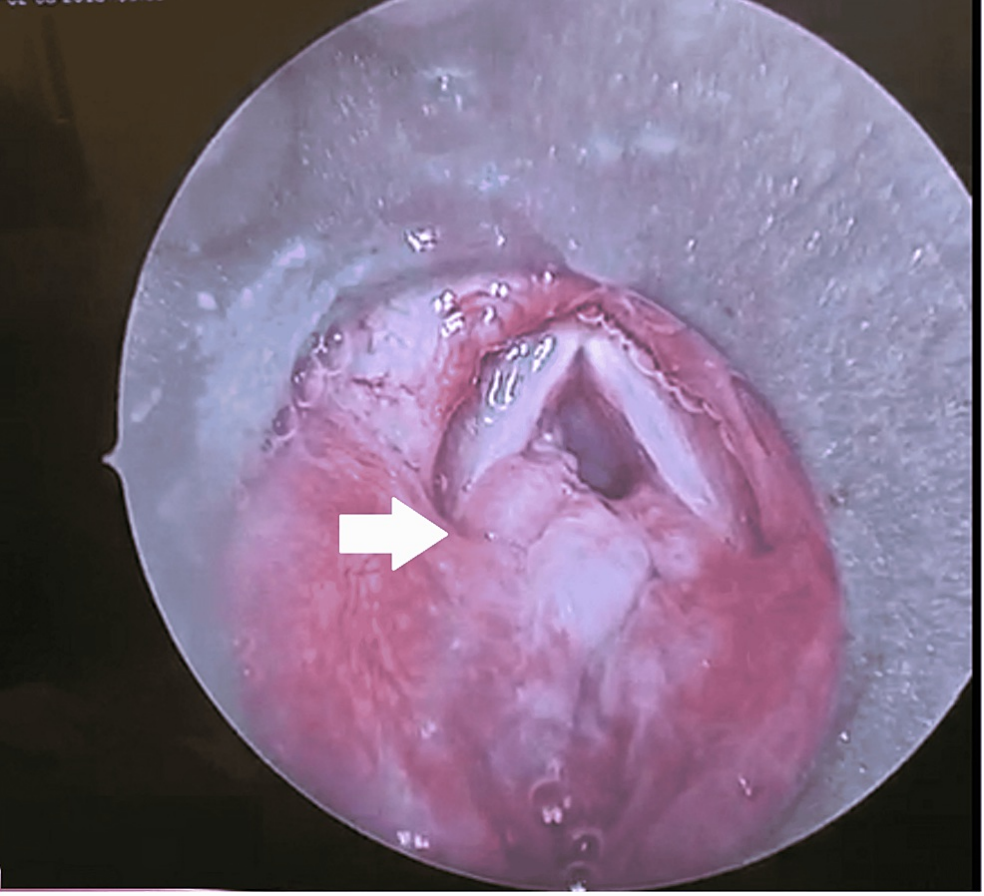
Endoscopic image captured intraoperatively showing a swelling over the posterior commissure extending inferiorly to the subglottic region (white arrow).

**Figure 2:**
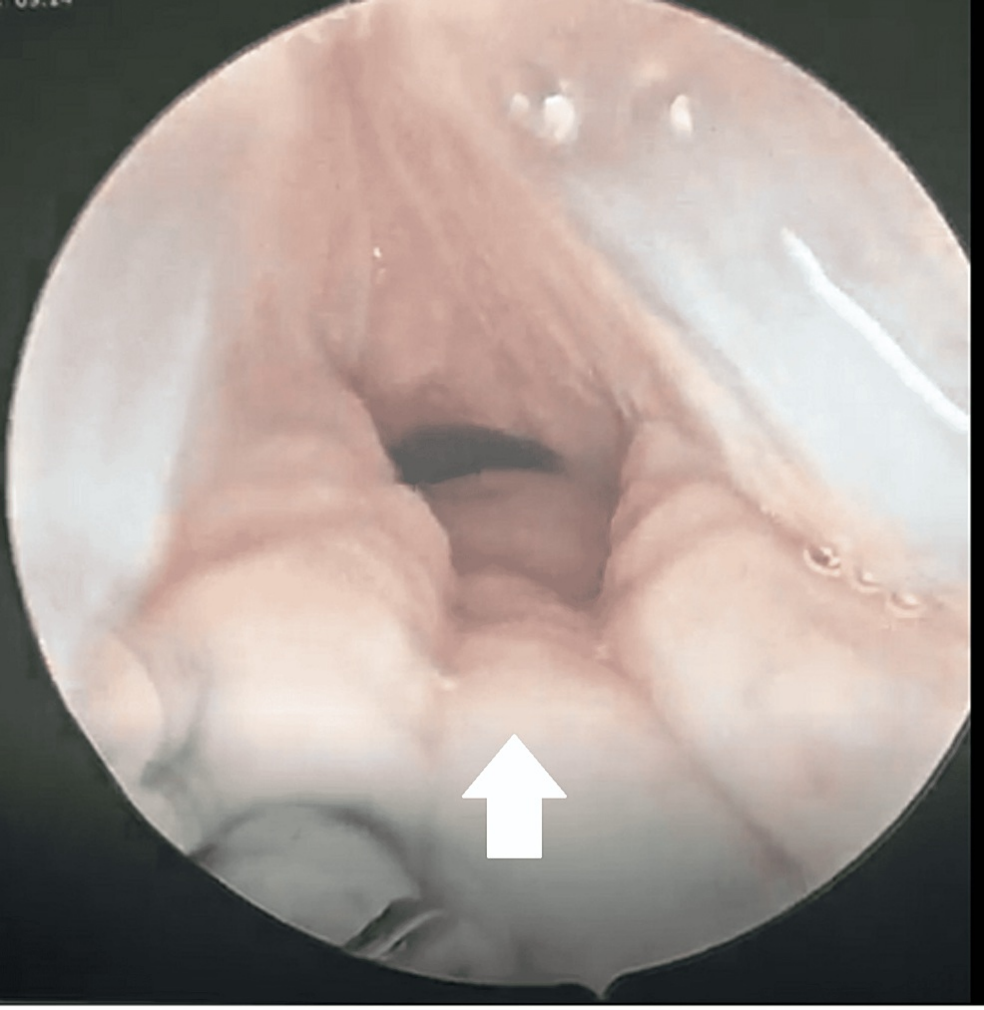
Endoscopic image captured intraoperatively showing the subglottic region that was edematous and collapsed (white arrow).

He underwent emergency tracheostomy with further investigation via computed tomography (CT) scan that showed severe stenosis at the subglottic area encompassing approximately a 4-cm segment. Right after stabilization, he was referred to our team for further management. Without delay, direct laryngoscopy was done under general anesthesia, which showed generalized edema of the larynx and trachea with an indurated swelling involving the arytenoids and posterior commissure (Figure 1). The subglottic region appeared collapsed and edematous with the tracheal ring poorly identified due to the localized edema (Figure 2).

Local corticosteroid injection was administered during the procedure. The postoperative provisional diagnosis was relapsing polychondritis. Tapering doses of oral steroids were thereby prescribed to the patient.

Upon the completion of the oral steroid dosage, a direct laryngoscopy procedure was repeated, and the findings remained unchanged. Biopsy taken during the second intervention remained inconclusive as it merely displayed atypical stromal cells with extensive crushed artifacts. A concomitant autoimmune screening was done, indicating positive ANA and high values of erythrocyte sedimentation rate (ESR) and C-reactive protein (CRP). The case was comanaged by the pediatric team, whereby their expert opinion ruled out the diagnosis of relapsing polychondritis. Instead, the child was treated for granulomatosis with polyangiitis, which is a systemic necrotizing vasculitis. The extractable nuclear antigen (ENA) test was found to be negative, thereby necessitating further referral to the pediatric rheumatology team in another tertiary center. However, the patient did not show improvements despite optimal immunosuppressant treatment.

Due to the nonresponsive nature of the condition, a third direct laryngoscopy and examination under anesthesia were performed. The repeated biopsy taken from the mass was confirmed to be an embryonal rhabdomyosarcoma. Meanwhile, CT scan and magnetic resonance imaging (MRI) of the neck done collectively displayed a heterogeneous mass seen in the neck, which extended into the trachea and retrosternal region, causing complete obstruction. The child was then referred to another tertiary center with oncology specialty.

## Discussion

Rhabdomyosarcomas are rarely found in the larynx, in which most of the cases of head and neck rhabdomyosarcomas occur in the orbit, nasopharynx, and nose [2,3]. A particular study undertaken over a period of 20 years reported that children presenting with head and neck rhabdomyosarcomas have a mean age of 5.3 years and a median age of four years. A male predilection is evident, with a male/female ratio of 1.7:1. The primary tumor sites and percentages are as follows: face/non-orbital sites, 18%; orbit/periorbital, 16%; nasal cavity/nose, 14%; lymph nodes, 12%; paranasal sinuses, 10%; parameningeal, 10%; parotid gland, 6%; neck, 6%; infratemporal fossa/zygoma, 2%; buccal mucosa, 2%; palate, 2%; and larynx, 2% [4]. The study has clearly indicated that the laryngeal region merely accounts for 2% of rhabdomyosarcomas, underlining the uncommon incidence of laryngeal rhabdomyosarcoma. The rarity of these lesions rendered them to be practically unconsidered during the process of obtaining a clinical differential diagnosis of laryngeal malignancies. Such a rarity inevitably misleads medical practitioners from reaching an accurate diagnosis.

The presenting symptoms may also aid in determining the provisional diagnosis, subsequent investigation selection, and further management. Typically, the most common presenting symptoms in laryngeal carcinoma consist of dysphagia, hoarseness of voice, and swelling in the neck. Airway compromise is a late presentation and merely occurs when the growth has considerably increased in size [5]. In this case, the symptoms presented by the patient were subtle, vague, and not typically seen in most laryngeal carcinoma patients. The imaging protocol for laryngeal pathology in children should include the following: ultrasonography as a quick and radiation-free imaging modality, magnetic resonance imaging for better assessment of the soft tissues, or computer tomography with contrast media that is widely available [6].

Certain serological markers (e.g., ANA) are commonly seen in autoimmune diseases such as rheumatoid arthritis, granulomatosis with polyangiitis, and relapsing polychondritis. However, several studies have shown that the antinuclear factor is significantly higher in the number of patients having a variety of malignant diseases compared to the normal control groups. The incidence of such positive tests is conclusively and significantly greater in the tumor group, allowing the conclusion that a malignant disease should be strongly suspected in the presence of consistently positive antinuclear antibodies [7].

The interpretation of the significance of autoantibodies in malignancies has remained controversial despite examples of autoantibodies commonly found in specific autoimmune diseases, as seen in the case we described. Some studies also highlighted the conclusion that a large number of autoantibodies found in malignancies do not recognize the autoantigens classically associated with the particular autoimmune disease [7,8]. These conflicting data are generally interpreted as an indication of autoantibody nonspecificity in malignancies. However, exceptions are known to occur; a relatively small but steadily broadening group of autoantibodies perceiving the same antigens has been reported in both cancer and autoimmune diseases alike [8,9].

In this case, suspicions rose as other tested autoantibodies specified for certain autoimmune diseases revealed negative outcomes, whereas the markers of inflammatory conditions such as ESR and CRP were high. This inevitably results in the dilemma of achieving an accurate diagnosis.

Eventually, the right diagnosis was made with the aid of a multidisciplinary approach and in consideration of other comanaging disciplines’ opinions and suggestions. The combination of multidisciplinary opinions and an adequate investigation and procedural steps taken allowed an accurate diagnosis to be reached. The management of rhabdomyosarcoma has shifted drastically from radical debilitating surgery to a less morbid treatment protocol, which is now typically focused on organ-sparing procedures supplemented with chemotherapy and/or radiotherapy. This paradigm shift is based on the multimodality recommendations made by the Intergroup Rhabdomyosarcoma Study Group (IRSG) [10]. Rhabdomyosarcoma of the larynx appears to be highly responsive to chemotherapy. However, the treatment responses of radiotherapy were variable and often unpredictable. Therefore, the optimal therapy for laryngeal rhabdomyosarcoma is a multimodal, multidisciplinary approach comprising surgery followed by chemotherapy and/or radiotherapy [11].

## Conclusions

We presented an unusual case of pediatric laryngeal embryonal rhabdomyosarcoma that poses various diagnostic and treatment dilemmas. One must always be vigilant and have a high index of suspicion, particularly in the case of patients presenting with vague and uncommon clinical presentation, as seen in the case reported here. Laryngeal embryonal rhabdomyosarcoma is highly responsive to combination chemotherapy and radiotherapy; therefore, recommendations are now focused on organ-sparing procedures supplemented with chemotherapy and/or radiotherapy.
